# Safety and Intranasal Retention of a Broad-Spectrum Anti-SARS-CoV-2 Monoclonal Antibody SA55 Nasal Spray in Healthy Volunteers: A Phase I Clinical Trial

**DOI:** 10.3390/pharmaceutics17010043

**Published:** 2024-12-31

**Authors:** Chaoying Hu, Yibo Zhou, Xing Meng, Jianhua Li, Jinxia Chen, Zhifang Ying, Xiaoliang Sunney Xie, Yaling Hu, Yunlong Cao, Ronghua Jin

**Affiliations:** 1 Phase I Clinical Trial Unit, Beijing Ditan Hospital, Capital Medical University, Beijing 100015, China; huchaoying@mail.ccmu.edu.cn (C.H.); zhouyibo@mail.ccmu.edu.cn (Y.Z.); 2 Clinical Research and Development Center, Sinovac Biotech Co., Ltd., Beijing 100085, China; mengx@sinovac.com; 3 Zhejiang Key Laboratory of Public Health Detection and Pathogenesis Research, Hangzhou 310051, China; jhli@cdc.zj.cn; 4 Clinical Research and Development Center, Sinovac Life Sciences Co., Ltd., Beijing 102601, China; chenjx0369@sinovac.com (J.C.); huyl@sinovac.com (Y.H.); 5 Respiratory Virus Vaccine, National Institutes for Food and Drug Control, Beijing 100061, China; yzf_0812@nifdc.org.cn; 6 Biomedical Pioneering Innovation Center (BIOPIC), Peking University, Beijing 100871, China; sunneyxie@pku.edu.cn; 7 Changping Laboratory, Beijing 102206, China

**Keywords:** SARS-CoV-2, monoclonal antibody, SA55 nasal spray, phase I clinical trial, broad-spectrum neutralizing antibody, intranasal administration

## Abstract

Background: A broad-spectrum anti-SARS-CoV-2 monoclonal antibody (mAb), SA55, is highly effective against SARS-CoV-2 variants. This trial aimed at demonstrating the safety, tolerability, local drug retention and neutralizing activity, systemic exposure level, and immunogenicity of the SA55 nasal spray in healthy individuals. Methods: This phase I, dose-escalation clinical trial combined an open-label design with a randomized, controlled, double-blind design. Healthy participants aged 18–65 years were enrolled and received a single dose of the SA55 nasal spray (1 mg or 2 mg) or multiple doses of SA55 nasal spray/placebo for 7 days (1 or 2 mg/dose, 3 or 6 doses/day). Safety monitoring was conducted throughout the study. Nasal swabs and venous blood samples were collected to analyze local drug concentration/neutralization, systemic exposure, and immunogenicity. Results: From 2 June to 11 August 2023, 80 participants were enrolled and received study intervention. The severity of adverse reactions (ADRs) reported during the study was mild in all cases, and all ADRs were laboratory test abnormalities without corresponding symptoms or vital signs. A total of 9 ADRs were reported, of which all were mild in severity. Overall ADR incidence rate was 16.67% (8/48) in single-dose groups and 4.17% (1/24) in multiple-dose groups. The nasal local drug concentration and neutralizing activity were generally stable within 4–8 h, with favorable neutralization activity against Omicron BF.7 and XBB strains. Conclusions: This study demonstrated favorable safety and tolerability of the SA55 nasal spray in healthy volunteers, exhibited satisfactory neutralizing activity against Omicron variants intranasally, and indicated low systemic toxicity risk.

## 1. Introduction

Since the end of 2019, the severe acute respiratory syndrome coronavirus 2 (SARS-CoV-2) has been spreading globally. On account of its widespread transmission, the World Health Organization (WHO) declared the situation a Public Health Emergency of International Concern on 30 January 2020 [1] and finally stated it as a pandemic of coronavirus disease 2019 (COVID-19) on 11 March 2020 [2]. During the COVID-19 epidemic, various COVID-19 vaccines and therapies were developed and implemented worldwide, making important contributions to the prevention and control of COVID-19 [3,4,5]. Neutralizing antibody (NAb) drugs against SARS-CoV-2 also showed a favorable effect on the prevention or treatment of COVID-19 by linking directly to the virus, thereby inhibiting further infection. The US Food and Drug Administration (FDA) approved the emergency use of several anti-SARS-CoV-2 antibody therapies during the pandemic, including single-antibody therapies, such as Bamlanivimab (Lilly) [6], Bebtelovimab (Lilly) [7], and Sotrovimab (GSK) [8], and dual-antibody combination therapies, such as Evusheld (Tixagevimab/Cilgavimab, AstraZeneca) [9], REGEN-COV (Casirivimab/Imdevimab, Regeneron) [10], and Bamlanivimab/Etesevimab (Lilly) [11].

However, as time has gone on, SARS-CoV-2 has undergone continuous evolution, leading to the emergence of numerous variants and thereby posing novel challenges in the prevention and treatment of COVID-19 [12,13,14,15,16,17,18,19,20,21]. New variants developed the capacity to evade immunity acquired through vaccination or previous SARS-CoV-2 infection, as well as NAb drugs against SARS-CoV-2. Current monoclonal antibody COVID-19 therapies authorized for emergency use authorization (EUA) target the receptor-binding domain (RBD) of the SARS-CoV-2 spike glycoprotein. Although combinations of monoclonal antibodies (antibody cocktails) have been developed to prevent potential escape by targeting multiple viral epitopes, the Omicron variants have been able to escape many Nab therapies [13,15,20,22,23]. Recently, the FDA has withdrawn all emergency use authorizations for previously launched COVID-19 neutralizing antibodies. Therefore, the development of broad-spectrum neutralizing antibodies has emerged as a crucial strategy in addressing the rapid mutation of SARS-CoV-2 globally.

Currently, NAb therapies against SARS-CoV-2 are primarily administered through intravenous infusion/intramuscular injection. However, the amount of drug that can reach the surface of upper respiratory mucosa, such as the nasal mucosa, is relatively limited. It accounts for approximately 1~2% of the blood drug concentration [24]. Given that nasal mucosa epithelial cells are the initial site of SARS-CoV-2 infection in the human body, poor local availability limits the effectiveness of NAb drugs in preventing SARS-CoV-2 infection. Consequently, several SARS-CoV-2 antibody drugs administrated intranasally are under development, which may allow for faster and better protection against SARS-CoV-2 infection and be more suitable for widespread use in emergency situations. Some NAb nasal spray products have entered the clinical development stage, including the 35B5 mAb nasal spray, which harbors broad neutralization to SARS-CoV-2 variants of concern [25]; A8G6, a combination of two monoclonal NAbs [26]; human IgG1 anti-SARS-CoV-2 antibody cocktail [27]; and SA58, a broad-spectrum anti-SARS-CoV-2 mAb [28,29]. Some of them showed favorable real-world effectiveness in the post-exposure prophylaxis of COVID-19 [26,28,29], offering encouragement for the development of similar drugs.

The SA55 nasal spray, a broad-spectrum anti-SARS-CoV-2 mAb, was identified from a large library containing 13,000 antibodies isolated from SARS-CoV-2-vaccinated SARS convalescents using high-throughput single-cell sequencing technology and yeast display technology [30]. It is highly resistant to mutations causing immune evasion to several earlier discontinued mAbs and has been shown to potently neutralize ACE2-utilizing sarbecoviruses, including circulating Omicron variants (i.e., BA.1, BA.2, BA.2.12.1, BA.3, BA.4/BA.5, BF.7, and BQ.1.1) in in vitro neutralizing assays and in animal challenge studies [18,19,20,21,30,31]. The passage of rVSV-SARS-CoV-2 pseudovirus shows its potential to avoid escape. Moreover, the stability of the SA55 nasal spray is favorable. Based on the findings from the interim stability studies, it can be preserved for a minimum of 9 months when stored at 5 °C ± 3 °C and for a minimum of 1 month when stored at 25 °C ± 2 °C. Compared with vaccines/drugs on the market, the SA55 nasal spray may have unique advantages and is expected to be an ideal choice as a short-acting prevention of SARS-CoV-2 infection.

In this phase I clinical trial, we aimed at demonstrating the safety, tolerability, nasal local drug concentration and neutralizing activity in the nasal cavity, systemic exposure level, and immunogenicity of the SA55 nasal spray in healthy individuals.

## 2. Materials and Methods

### 2.1. Study Design

This phase I clinical trial combined an open-label design with a randomized, controlled, double-blind design to evaluate the safety, nasal local drug concentration and nasal local neutralizing activity, systemic exposure level, and immunogenicity of the SA55 nasal spray in healthy adults (Clinical Trials Registration: NCT06048393). This study was approved by the Ethics Committee of Beijing Ditan Hospital, Capital Medical University (Reference No. DTEC-YW2023-009-05). The dose escalation consists of two parts. The first part was an open-label design, in which participants received a single dose of the SA55 nasal spray at either a 1 mg or 2 mg dosage. The second part was conducted as a randomized, controlled, double-blind design, in which participants were assigned to receive the SA55 nasal spray or placebo at different dosages and with different frequencies for 7 days. The ratio of participants receiving the SA55 nasal spray to those receiving placebo was 3:1 within each group.

### 2.2. Participants

Healthy participants aged 18–65 years were enrolled in this trial. The main exclusion criteria were a history of allergy to any component of the investigational product or to inhaled allergens; poorly controlled chronic diseases or history of severe illnesses; having previously received an anti-SARS-CoV-2 antibody/COVID-19 nasal spray vaccine within the past 180 days; and having been vaccinated with a COVID-19 vaccine or having an infection history of SARS-CoV-2 within the previous 90 days.

### 2.3. Randomization and Masking

The first part of this study (single-dose) was randomized with an open-label design. In this part, participants of group A (1 mg, single dose) and group B (2 mg, single dose) were randomly allocated to 4 groups (A1, A2, A3, A4 or B1, B2, B3, and B4) at a 1:1:1:1 ratio, with 6 participants in each group. The second part (multiple dose) of this study was randomized with a controlled, double-blind design. In this part, 8 participants in each group (groups C, D, E, and F) were allocated to receive SA55 or placebo in a 3:1 ratio. In all groups of part 2, the first sampling site (right nasal cavity or left nasal cavity) was randomized as well. Participants in each group were randomly assigned at a 1:1 ratio to provide the 1st nasal swab in the right nasal cavity or left nasal cavity. SAS 9.4 version was used.

### 2.4. Procedures

The SA55 nasal spray was formulated into prefilled sprayers (containing 20 sprays per bottle), with each milliliter containing 5 mg of SA55. The placebo did not contain any neutralizing antibodies, and all other components were identical to the investigational drug. Each administration involves one or two sprays in each nostril, resulting in a total of 1 or 2 mg per application (i.e., a total of 2 or 4 sprays, with 0.5 mg per spray).

The inclusion of participants was conducted in a dose-escalating order, where the higher-dose group was enrolled after the completion of the safety assessment in the lower-dose group without significant safety issues observed. Since the daily dosages for participants in groups D and E were the same, the enrollment of these two groups was carried out simultaneously. All participants underwent safety monitoring throughout the study. Nasal swabs and venous blood samples were collected to analyze local drug concentration, local neutralizing activity, systemic exposure, and immunogenicity (Table S1). For single-dose groups (groups A and B), to avoid the impact of repeated swabbing on local drug concentration assessment at different timepoints, participants in groups A and B only provided nasal swabs at two timepoints after nasal spray administration. In this case, each participant performed only one nasal swab per nostril.

### 2.5. Outcomes

The primary endpoints were the incidence of adverse events and serious adverse events during the study period. The secondary endpoints were local (intranasal) drug concentrations, local (intranasal) NAb levels, serum concentrations, and anti-drug antibody (ADA) levels at different timepoints after administration.

### 2.6. Local Concentration, Local NAb Titer, Systemic Exposure, and Immunogenicity Evaluation

For local concentration and NAb titer evaluation, nasal swabs were collected at various timepoints before and after administration. The specific nasal swab sampling timepoints are listed in Table S1. Mass spectrometry was used to analyze the drug concentration, and a cell cytotoxicity assay was used to analyze the local neutralizing activity against SARS-CoV-2. For systemic exposure and immunogenicity evaluation, venous blood samples were collected at different timepoints before and after administration. The SA55 concentration and ADA levels in serum were measured at different timepoints (Table S1). Liquid chromatography–tandem mass spectrometry (LC-MS/MS) was used to analyze the SA55 concentration (liquid chromatography: ExionLC AD Pump, AB SCIEX, Framingham, MA, U.S.; mass spectrometry: TRIPLE QUAD 6500^+^ AB SCIEX, woodlands, Singapore), and an ELISA assay (Meso Scale Discovery Inc. MESO QUICKPLEX SQ120, MSD, Rockville, MD, USA) was used to analyze the ADA levels.

### 2.7. Statistical Analysis

Analysis of primary endpoints was based on the safety set, which included all participants who were randomized and received at least one dose of study intervention. The 95% bilateral confidence intervals of percentage were calculated by the Clopper–Pearson method. All statistical analyses were performed using SAS 9.4 or higher version of statistical software.

## 3. Results

### 3.1. Characteristics of Study Population

**Figure 1:**
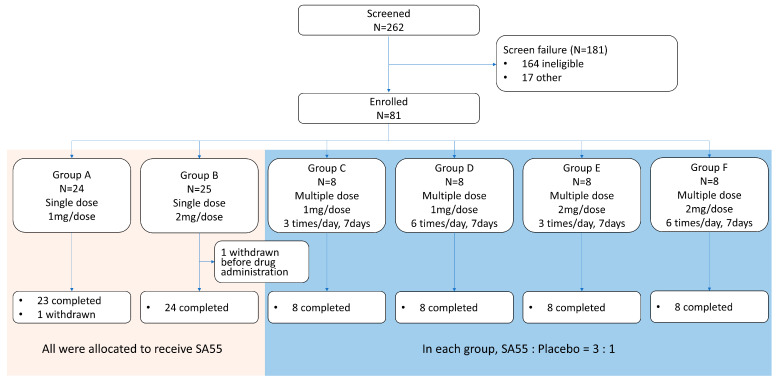
Participant disposition. A total of 80 participants received single or multiple dose(s) of SA55 nasal spray in groups A–F.

From 2 June to 11 August 2023, a total of 262 healthy adults were screened, and 81 were enrolled in the trial. Among them, 1 participant in group B dropped out due to abnormal vital signs before the study intervention, and 80 participants completed the study intervention according to protocol. One participant in group A withdrew after completing drug administration (lost to follow-up). A total of 79 participants completed all follow-up visits (Figure 1). The demographic characteristics were generally consistent among study groups. The average age of participants was 33.2 years in single-dose groups (groups A/B, *n*= 48) and 38.7 years in multiple-dose groups (groups C/D/E/F, *n* = 32) (Table 1).

### 3.2. Safety

The severity of ADRs reported during the study was mild in all cases, and all ADRs were laboratory test abnormalities without any corresponding symptoms or vital signs. The total ADR incidence rate was 16.67% (8/48) for single-dose groups and 4.17% (1/24) for multiple-dose groups (among the 24 participants who received the investigational drug). Only one case of blood bilirubin increase was reported in group C (Table 2).

With the exception of a grade 2 ADR (neutrophil count decrease), all other reactions were grade 1. No ADR or SAE of grade 3 or higher severity was reported. No increase in ADR incidence rates was observed with dose escalation, suggesting that there was no dose-related risk. The highest dosage administered to the multiple-dose group was 2 mg/dose, 6 doses/day, which demonstrated favorable safety and tolerance without any nasal irritation.

### 3.3. The Local Drug Content and Neutralizing Activity

Since a nasal spray is gradually cleared from nasal mucosa by the movement of nasal cilia, it was necessary to evaluate the local retention of the SA55 nasal spray on nasal mucosa to determine the frequency of administration. Participants received nasal swab sampling at different timepoints after medication to assess the local drug concentration and neutralization.

**Figure 2:**
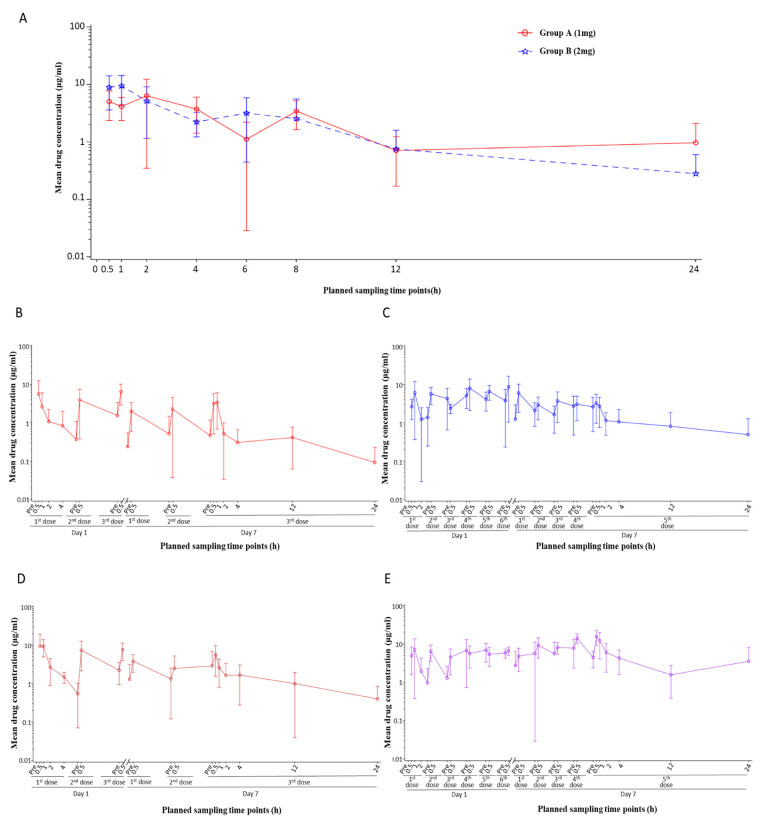
Intranasal local drug concentration after single/multiple-dose(s) administration. For single-dose groups, intranasal local drug concentration after nasal spray administration in group A (1 mg dosage) and group B (2 mg dosage) (**A**) was assessed at different timepoints within 24 h. For multiple-dose groups, intranasal local drug concentration was assessed at different timepoints pre-/post-dose on day 1 and day 7/day 8 for group C (1 mg/time, 3 doses/day) (**B**), group D (1 mg/time, 6 doses/day) (**C**), group E (2 mg/time, 3 doses/day) (**D**), and group F (2 mg/time, 6 doses/day) (**E**). Geometric mean of drug concentrations with error bars indicating the SD (standard deviation) is plotted.

After a single dose with different dosages, the nasal local drug concentration in groups A and B peaked within 2 h after administration, which was 6.37 μg/mL and 9.42 μg/mL, respectively. From 2 h after administration, the nasal local drug concentration levels of groups A and B were similar and relatively stable within 4 h, gradually decreased with time, and diminished to a minimal level 24 h after administration (0.97 μg/mL and 0.23 μg/mL) (Figure 2A).

**Figure 3:**
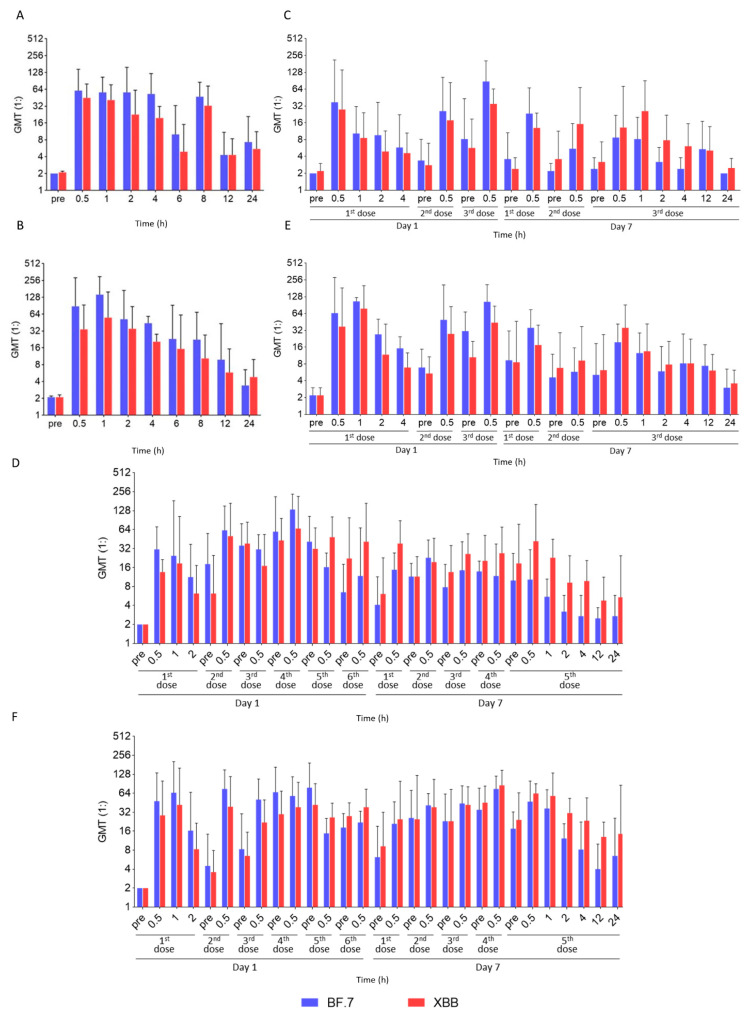
Intranasal neutralizing capacity after single/multiple-dose(s) administration. For single-dose groups, intranasal local neutralization against Omicron BF.7 variant and XBB variant of group A (1 mg dosage) (**A**) and group B (2 mg dosage) (**B**) was assessed at different timepoints within 24 h after nasal spray administration. For multiple-dose groups, intranasal local neutralization against Omicron BF.7 variant and XBB variant was assessed at different timepoints pre-/post-dose on day 1 and day 7/day 8 for group C (1 mg/time, 3 doses/day) (**C**), group D (1 mg/time, 6 doses/day) (**D**), group E (2 mg/time, 3 doses/day) (**E**), and group F (2 mg/time, 6 doses/day) (**F**). Geometric mean drug concentrations with error bars indicating the 95% CI (confidence interval) are plotted.

The results of nasal local neutralization showed that the level of local NAb rapidly increased after administration, with favorable neutralization activity against Omicron BF.7 and XBB strains. The peak levels of local neutralization activity were observed within 1 h after single administration in groups A and B (GMT against BF.7 strain: 60.5 for group A, 142.4 for group B; GMT against XBB strain: 44.9 for group A, 55.4 for group B) (Figure 3A,B). Echoing the results of local drug concentration, the neutralization activity levels between groups receiving different single dosages were similar after 1 h. Within 8 h after administration, the overall neutralization activity was favorable and stable (the GMT was generally higher than 16 for both groups against BF.7 within 8 h) and then gradually declined, suggesting that a dose interval within 8 h should be capable of continuous protection against new SARS-CoV-2 exposure intranasally.

In the multiple-dose groups, the local peak drug concentration occurred 30 min after each administration, and the local trough drug concentration was detected before each administration. Both peak and trough local concentrations increased with the increase in the single dosage and daily dosing frequency. When comparing group D (1 mg—6 doses/day) with group E (2 mg—3 doses/day), it was found that the ranges of local peak drug concentration were similar (2.38~8.75 μg/mL vs. 2.47~9.34 μg/mL) in both groups, but the range of local trough drug concentration in group D was higher than that in group E (1.25~5.20 μg/mL vs. 0.54~2.88 μg/mL) (Figure 2B–E). Similar results were observed in the local neutralization assessment: the nasal swab eluent collected before and after each administration generally had neutralizing activity against SARS-CoV-2, and neutralizing activity levels tended to be more stable in group D than in group E. These results suggest that when the total daily dosage remained constant, increasing the frequency of daily administration was more beneficial to stabilizing the nasal local drug concentration at a relatively high level, providing better protection (Figure 3C–F).

Furthermore, no accumulation phenomenon was observed during the multiple-dosing stage. The local drug concentration peak after each administration in the multiple-dose groups was consistent with that observed after corresponding single-dose administration. There was no significant increase in local concentration after multiple dosing compared with that in single-dosing groups. Additionally, no increase in local drug concentration was observed on day 7 compared with that on day 1. Similarly, the local drug concentration before each administration (trough concentration) also showed this trend. Therefore, when the SA55 nasal spray was administered continuously for 7 days at a dosage of 1 mg/2 mg, administered at either 3 doses/day or 6 doses/day, no obvious accumulation phenomenon was observed in the nasal cavity.

### 3.4. System Exposure and Immunogenicity

System exposure (serum drug concentration) was evaluated in all participants receiving different dosages of SA55. For groups A–E, the serum concentrations of SA55 were all below the lower limit of quantification at each timepoint. In group F (2 mg—6 doses/day), trace amounts of SA55 were detected in the serum at three timepoints (before the first dose on D7 and 30 min after the last dose on D7 and on D8) in a total of four participants. The individual serum drug concentrations ranged between 4.03 ng/mL and 5.05 ng/mL, close to the lower limit of quantification (4 ng/mL). The mean drug blood concentrations at each timepoint were between 1.38 ng/mL and 3.02 ng/mL (Figure S1). The ADA level after dosing was also evaluated within 28 days after 1st dose in multiple-dosing groups, suggesting that SA55 nasal spray does not induce ADA after administration.

## 4. Discussion

Like many airborne diseases, SARS-CoV-2 is primarily transmitted through the mucous epithelial cells of the upper respiratory tract (nasal, mouth, and oropharynx) by inhaling droplets/aerosols including infectious SARS-CoV-2 particles [32]. An intranasal spray of neutralizing antibodies may offer a more effective means of preventing viral entry directly, which may offer potential short-term protection in high-exposure environments, such as business travels, large-scale conventions, and hospital visits, or which may reduce the risk of respiratory tract infection after suspected exposure, including SARS-CoV-2 infection. At present, most prophylactic antibody drugs, including previously authorized SARS-CoV-2 antibody treatments/prevention by EUA, are administrated via intravenous or intramuscular injection [33], which may be inconvenient to users due to the invasive administration. As an intranasal spray, the non-invasive administration may lead to less hesitancy, especially among children, and may result in better medication compliance. Therefore, the development of monoclonal antibody nasal sprays serves a dual purpose: it not only helps to prevent SARS-CoV-2 infection but also presents a swift and powerful alternative for prevention against other airborne diseases, making it an ideal solution for emergency scenarios. Moreover, the development of prophylactic antibody nasal sprays also provides insights into the local distribution and systemic exposure of antibody drugs when administered intranasally, laying the groundwork for the development of therapeutic antibodies targeting respiratory diseases.

This first-in-human trial highlights the favorable safety and local retention profile of the SA55 nasal spray and also shows satisfactory local neutralization levels. Notably, no obvious toxic effects, nasal irritation, or serious adverse events occurred during the trial, and the severity of ADRs reported during the study was all mild. All ADRs were laboratory test abnormalities without any corresponding symptoms or vital signs.

Local concentration/neutralization profiles after SA55 administration were evaluated in this trial to guide the medication regimen for the SA55 nasal spray in future studies. The variation trend of local (nasal) drug concentration and neutralization capacity was consistent after administration. Results suggest that, to provide continuous protection for all participants, the dose interval should be proposed within 4–8 h, considering individual differences. The local antibody concentration and neutralization capacity in the nasal cavity were relatively stable within 4 h with a single dose of 1 mg or 2 mg and then decreased gradually. The local neutralization potency against Omicron variants was favorable (GMTs were generally higher than 16) within 8 h, indicating adequate protection. In addition, in multiple-dose groups, increasing the dosage for a single administration or increasing the administration frequency could both help to prolong local drug retention. Upon analysis of local drug neutralization potency, it was observed that rather than increasing the dosage of each administration, increasing the frequency of administrations can more effectively maintain the stability of local drug concentration, thereby possibly achieving superior protective effects.

One limitation of this trial is the small number of participants, typical of phase I trials. Another limitation of this study is that, in order to accurately evaluate the local pharmacokinetics after a single dose and to eliminate the underestimation of local drug residue caused by multiple sampling after a single dose in the same place, participants in the single-dose group were only sampled once from each nasal cavity after administration. Therefore, the drug concentration–time curve was plotted based on data from different participants, making it impossible to calculate pharmacokinetic parameters.

In conclusion, this study demonstrated the favorable safety and tolerability of the SA55 nasal spray in healthy volunteers and satisfactory intranasal neutralizing activity against Omicron variants after administration. Furthermore, minimal systemic exposure was observed in the highest-dosage group, and no drug-related anti-drug antibodies were detected, indicating a low risk of systemic toxicity.

## Figures and Tables

**Table 1:** Baseline characteristics of participants who received study intervention.

Items	Single Dose			Multiple Dose					
	Group A 1 mg	Group B 2 mg	Total	Group C 1 mg/dose 3 Doses/Day	Group D 1 mg/dose 6 Doses/Day	Group E 2 mg/dose 3 Doses/Day	Group F 2 mg/dose 6 Doses/Day	Placebo	Total
	*n* = 24	*n* = 24	*n* = 48	*n* = 6	*n* = 6	*n* = 6	*n* = 6	*n* = 8	*n* = 32
Age (years) Mean (SD)	31.2 (6.8)	35.3 (9.0)	33.2 (8.2)	35.8 (10.8)	35.7 (5.8)	41.8 (8.1)	44.0 (7.3)	36.8 (7.5)	38.7 (8.3)
Gender n (%)									
Male	15 (62.50)	19 (79.17)	34 (70.83)	4 (66.67)	4 (66.67)	3 (50.00)	3 (50.00)	6 (75.00)	20 (62.50)
Female	9 (37.50)	5 (20.83)	14 (29.17)	2 (33.33)	2 (33.33)	3 (50.00)	3 (50.00)	2 (25.00)	12 (37.50)
Ethnicity n (%)									
Han	23 (95.83)	21 (87.50)	44 (91.67)	6 (100.00)	5 (83.33)	6 (100.00)	6 (100.00)	6 (75.00)	29 (90.63)
Other	1 (4.17)	3 (12.50)	4 (8.33)	0 (0.00)	1 (16.67)	0 (0.00)	0 (0.00)	2 (25.00)	3 (9.38)
Height (cm) Mean (SD)	167.48 (10.58)	167.89 (7.98)	167.68 (9.27)	163.97 (5.79)	167.25 (8.72)	163.47 (8.28)	161.97 (6.61)	165.05 (8.64)	164.38 (7.46)
Weight (kg) Mean (SD)	64.73 (11.19)	64.96 (9.52)	64.85 (10.28)	65.40 (6.08)	61.53 (8.01)	63.63 (8.58)	65.49 (7.41)	68.98 (8.70)	65.26 (7.81)
BMI (kg/m^2^) Mean (SD)	22.93 (2.10)	23.00 (2.42)	22.96 (2.24)	24.42 (2.93)	21.92 (1.19)	23.73 (1.53)	24.95 (2.21)	25.34 (2.42)	24.15 (2.37)

**Table 2:** Adverse events of participants after administration of nasal spray by group.

AR Terms N (%)	Single Dose		Multiple Dose				
	Group A 1 mg	Group B 2 mg	Group C 1 mg/dose 3 Doses/Day	Group D 1 mg/dose 6 Doses/Day	Group E 2 mg/dose 3 Doses/Day	Group F 2 mg/dose 6 Doses/Day	Placebo
	*n* = 24	*n* = 24	*n* = 6	*n* = 6	*n* = 6	*n* = 6	*n* = 8
Total AR	4 (16.67)	4 (16.67)	1 (16.67)	0 (0.00)	0 (0.00)	0 (0.00)	0 (0.00)
Blood bilirubin increased	2 (8.33)	4 (16.67)	1 (16.67)	0 (0.00)	0 (0.00)	0 (0.00)	0 (0.00)
Neutrophil count decreased	1 (4.17)	0 (0.00)	0 (0.00)	0 (0.00)	0 (0.00)	0 (0.00)	0 (0.00)
Leukocyte count increased	1 (4.17)	0 (0.00)	0 (0.00)	0 (0.00)	0 (0.00)	0 (0.00)	0 (0.00)

## Data Availability

The data that support the findings of this study are available from the corresponding author, R.J., upon reasonable request.

## Supplementary Materials

The following supporting information can be downloaded at: https://www.mdpi.com/article/10.3390/pharmaceutics17010043/s1, Figure S1: Blood concentration of SA55 in Group F at different timepoints after administration; Table S1: Study design of dosing regimen and sample collection timepoints.
