# Supplementary material for: Safety and Intranasal Retention of a Broad-Spectrum Anti-SARS-CoV-2 Monoclonal Antibody SA55 Nasal Spray in Healthy Volunteers: A Phase I Clinical Trial
Source: Pharmaceutics. 2024 Dec 31;17(1):43. doi: 10.3390/pharmaceutics17010043 (PMC11768346; doi:10.3390/pharmaceutics17010043)
Supplement: Supplementary file 1 [file pharmaceutics-17-00043-s001.zip › pharmaceutics-3299666-supplementary figure S1.pdf]

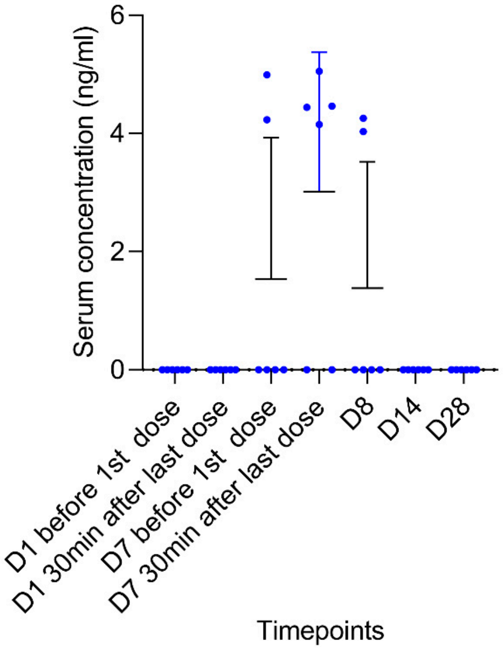

Supplementary Figure S1. Blood concentration of SA55 in Group F at different timepoints after administration.
