# Supplementary material for: Safety and Intranasal Retention of a Broad-Spectrum Anti-SARS-CoV-2 Monoclonal Antibody SA55 Nasal Spray in Healthy Volunteers: A Phase I Clinical Trial
Source: Pharmaceutics. 2024 Dec 31;17(1):43. doi: 10.3390/pharmaceutics17010043 (PMC11768346; doi:10.3390/pharmaceutics17010043)
Supplement: Supplementary file 1 [file pharmaceutics-17-00043-s001.zip › pharmaceutics-3299666-Supplementary Table S1.pdf]

Supplementary Table S1. Study design of dosing regimen and sample collection timepoints

Stage	Days	Group	Intervention	Dosage	Dose frequency	Sample size	Nasal swab collection time	Blood sample collection time
Single-dose	1	A1	SA55	1mg/dose	1doses/day	6	Pre-dose, 30min/6h post-dose	
		A2				6	Pre-dose, 1h/8h post-dose	
		A3				6	Pre-dose, 2h/12h post-dose	
		A4				6	Pre-dose, 4h/24h post-dose	**For system exposure assessment:**<br>Pre-dose, 30min/4h/12h/24h post-dose, D8
		B1	SA55	2mg/dose		6	Pre-dose, 30min/6h post-dose	
		B2				6	Pre-dose, 1h/8h post-dose	
		B3				6	Pre-dose, 2h/12h post-dose	
		B4				6	Pre-dose, 4h/24h post-dose	**For system exposure assessment:**<br>Pre-dose, 30min/4h/12h/24h post-dose, D8
**Subtotal**						48		
Multiple-dose#	7	C	SA55	1mg/dose	3doses/day	6	D1: before 1st dose, 30min/1h/2h/4h after 1st dose, before 2nd/3rd dose, 30min after 2nd/3rd dose;<br>D7: before each dose, 30min after each dose, and 30min/1h/2h/4h/12h/24h after last dose;	**For system exposure assessment:**<br>D1: before 1st dose, 30min after last dose;<br>D7: before 1st dose, 30min after last dose, D8, D14, D28;<br><br>**For ADA assessment:** before 1st dose of D1, D14, D28
			Placebo			2		
		D	SA55	1mg/dose	6doses/day *	6	D1: before 1st dose, 30min/1h/2h after 1st dose, before 2nd/3rd dose, 30min after 2nd/3rd dose;<br>D7: before each dose, 30min after each dose, and 30min/1h/2h/4h/12h/24h after last dose;	
			Placebo			2		
		E	SA55	2mg/dose	3doses/day	6	D1: before 1st dose, 30min/1h/2h/4h after 1st dose, before 2nd/3rd dose, 30min after 2nd/3rd dose;<br>D7: before each dose, 30min after each dose, and 30min/1h/2h/4h/12h/24h after last dose;	
			Placebo			2		
		F	SA55	2mg/dose	6doses/day *	6	D1: before 1st dose, 30min/1h/2h after 1st dose, before 2nd/3rd dose, 30min after 2nd/3rd dose;<br>D7: before each dose, 30min after each dose, and 30min/1h/2h/4h/12h/24h after last dose;	
			Placebo			2		
**Subtotal**						32		
**Total**						80		

* Considering the feasibility of multi-sampling operation after the last dose, participants who need to receive 6 doses per day (group D and F) only received 5 doses on D7 to avoid sampling at late hours. Sampling after the last dose on D7 was conducted at the corresponding time point after the 5th dose on D7.
